# Lipoprotein(a) has no major impact on calcification activity in patients with mild to moderate aortic valve stenosis

**DOI:** 10.1136/heartjnl-2021-319804

**Published:** 2021-09-30

**Authors:** Yannick Kaiser, Nick S Nurmohamed, Jeffrey Kroon, Hein J Verberne, Evangelos Tzolos, Marc R Dweck, Aernout G Somsen, Benoit J Arsenault, Erik S G Stroes, Kang H Zheng, S Matthijs Boekholdt

**Affiliations:** 1 Department of Vascular Medicine, Amsterdam UMC, University of Amsterdam, Amsterdam Cardiovascular Sciences, Amsterdam, The Netherlands; 2 Department of Cardiology, Amsterdam UMC, Vrije Universiteit, Amsterdam Cardiovascular Sciences, Amsterdam, The Netherlands; 3 Department of Experimental Vascular Medicine, Amsterdam UMC, University of Amsterdam, Amsterdam Cardiovascular Sciences, Amsterdam, The Netherlands; 4 Department of Radiology and Nuclear Medicine, Amsterdam UMC, University of Amsterdam, Amsterdam, The Netherlands; 5 British Heart Foundation Centre for Cardiovascular Science, University of Edinburgh, Edinburgh, UK; 6 Cardiology Centers of the Netherlands, Amsterdam, The Netherlands; 7 Centre de Recherche de l'Institut Universitaire de Cardiologie et de Pneumologie de Québec, Québec Canada; 8 Department of Cardiology, Amsterdam UMC, University of Amsterdam, Amsterdam Cardiovascular Sciences, Amsterdam, The Netherlands

**Keywords:** aortic valve stenosis, positron emission tomography computed tomography

## Abstract

**Objective:**

To assess whether patients with aortic valve stenosis (AS) with elevated lipoprotein(a) (Lp(a)) are characterised by increased valvular calcification activity compared with those with low Lp(a).

**Methods:**

We performed ^18^F-sodium fluoride (^18^F-NaF) positron emission tomography/CT in patients with mild to moderate AS (peak aortic jet velocity between 2 and 4 m/s) and high versus low Lp(a) (>50 mg/dL vs <50 mg/dL, respectively). Subjects were matched according to age, gender, peak aortic jet velocity and valve morphology. We used a target to background ratio with the most diseased segment approach to compare ^18^F-NaF uptake.

**Results:**

52 individuals (26 matched pairs) were included in the analysis. The mean age was 66.4±5.5 years, 44 (84.6%) were men, and the mean aortic valve velocity was 2.80±0.49 m/s. The median Lp(a) was 79 (64–117) mg/dL and 7 (5–11) mg/dL in the high and low Lp(a) groups, respectively. Systolic blood pressure and low-density-lipoprotein cholesterol (corrected for Lp(a)) were significantly higher in the low Lp(a) group (141±12 mm Hg vs 128±12 mm Hg, 2.5±1.1 mmol/L vs 1.9±0.8 mmol/L). We found no difference in valvular ^18^F-NaF uptake between the high and low Lp(a) groups (3.02±1.26 vs 3.05±0.96, p=0.902). Linear regression analysis showed valvular calcium score to be the only significant determinant of valvular ^18^F-NaF uptake (β=0.63; 95% CI 0.38 to 0.88 per 1000 Agatston unit increase, p<0.001). Lp(a) was not associated with ^18^F-NaF uptake (β=0.17; 95% CI −0.44 to 0.88, p=0.305 for the high Lp(a) group).

**Conclusion:**

Among patients with mild to moderate AS, calcification activity is predominantly determined by established calcium burden. The results do not support our hypothesis that Lp(a) is associated with valvular ^18^F-NaF uptake.

## Introduction

Aortic valve stenosis (AS) is one of the most frequent cardiovascular diseases in the Western world.^1^ Although risk factors for AS are similar to those for atherosclerosis, previous randomised trials with statins or renin-angiotensin-aldosterone inhibitors have not been able to influence the progression of AS.^2^ Consequently, current management revolves around a wait-and-see approach until the development of severe AS warrants surgical or transcatheter valve replacement.^3^

Lipoprotein(a) (Lp(a)) has been established as an independent, likely causal risk factor for incident AS.^4–6^ Interventions aimed at lowering Lp(a), including proprotein convertase subtilisin/kexin type 9 inhibitors and apolipoprotein(a) antisense therapies, may be effective in delaying or preventing AS progression.^7^ However, the optimal timing of medical intervention in AS remains unresolved. The failure of previous statin trials to delay haemodynamic progression is often attributed to initiation at a disease stage too advanced to be amenable for intervention, at which point other disease mechanisms drive disease progression independent of hypercholesterolaemia.

Valvular ^18^F-sodium fluoride (NaF) uptake assessed by positron emission tomography (PET)/CT is robustly correlated with progression of calcification.^8^ A recent post-hoc analysis of longitudinal imaging studies reported that elderly patients with advanced AS and higher Lp(a) levels were characterised by higher valvular uptake of ^18^F-NaF and faster disease progression.^9^ These data support the concept of lowering Lp(a) in patients with established AS to mitigate disease progression. Whether earlier intervention in the disease process could be beneficial remains unexplored.

In the current study, we hypothesised that patients with mild to moderate AS and significantly elevated Lp(a) levels (>50 mg/dL) are characterised by a higher degree of valvular calcification activity than those with lower Lp(a) levels. As such, we performed a matched case–control study where we assessed valvular calcification activity by using ^18^F-NaF PET/CT in patients with mild to moderate AS with higher versus lower Lp(a) levels.

## Methods

### Patient selection

Participants were recruited from a biobank, consisting of 190 patients with mild to moderate AS who were previously recruited from the cardiology outpatient clinics of the Amsterdam University Medical Centers and Cardiologie Centra Nederland. Subjects were eligible for the study if they were aged between 50 and 80 years and had a peak aortic jet velocity between 2 and 4 m/s. Exclusion criteria were severe AS (peak aortic jet velocity >4 m/s), aortic valve area <1.0 cm^2^, history of radiotherapy of the thorax, history of rheumatic fever, estimated glomerular filtration rate <30 mL/min, hyperparathyroidism, Paget’s disease and/or any contraindication to PET/CT imaging. All eligible patients with high Lp(a) (>50 mg/dL) were asked to participate, who were then matched to controls with low Lp(a) levels according to sex, age, aortic valve morphology and peak aortic jet velocity. Detailed questionnaires regarding demographics, general health status, medical history, family history, risk factors, medication, blood pressure, height and weight were taken during the biobank study visit. Echocardiography was performed within 6 months of PET/CT imaging. All subjects gave written informed consent.

### Laboratory measurements

Baseline blood samples including Lp(a) measurements were available from a previous biobank study. Haematology, chemistry and lipid panels were determined according to standardised operating procedures in a core laboratory. Low-density-lipoprotein cholesterol (LDL-C) was calculated using the Friedewald formula.^10^ Lp(a) was measured in all patients on serum samples using the kringle IV type 2 number independent Randox immunoassay (Randox Laboratories, UK). We corrected LDL-C for Lp(a) by subtracting 0.15×Lp(a) mass from the LDL-C mass.^11^

### Echocardiography

Echocardiography was performed by the same research cardiologist if not performed within 6 months prior to PET/CT imaging, using a dedicated echocardiography device (Vivid E95, GE Healthcare, Chicago, USA). The aortic valve-specific echocardiogram included determination of aortic valve morphology, peak aortic jet velocity, transvalvular gradients and aortic valve area.

### Image acquisition and analysis

ECG-gated ^18^F-NaF PET and contrast-enhanced CT angiography were performed using a hybrid scanner (Biograph mCT, Siemens, Medical Systems, Erlangen, Germany) 60 min after infusion of 125 MBq ^18^F-NaF. If resting heart rate was >65 beats per minute, metoprolol 50 or 100 mg was administered according to clinical protocols. PET data were reconstructed using the Siemens Ultra HD reconstruction algorithm with correction for dead time, attenuation, random coincidences and scatter. ^18^F-NaF PET/CT images were assessed using FusionQuant V.1.20.05.14 (Cedars-Sinai, California, USA) using a standardised method.^12 13^ After fusing diastolic PET images with the contrast-enhanced CT, an en-face view was established and the PET window level adjusted to allow visual determination of the point of maximal specific valvular uptake. Automated correction for cardiac motion is possible and fully integrated within FusionQuant, and this was performed with an anatomically guided registration algorithm according to previously published methods.^14^ Briefly, a spherical region of interest was drawn to include the entire aortic valve. A non-linear registration algorithm, radially constrained around the aortic valve, was used to align PET images to the diastolic gate. The motion-corrected gates were then summed to form a motion-free image containing all the PET counts. A three-dimensional polyhedron, 6 mm in height and contoured to the valve frame, was centred on this point and formed the volume of interest. ^18^F-NaF blood pool uptake was measured in the right atrium with a cylinder 10 mm in radius by 10 mm in height. Where present, pacing leads in the right atrium were excluded. In the control cohort, the valvular region of interest was centred on the valve leaflets in the z-axis, ensuring the lower border included the leaflet bases. The aortic valve maximum target to background ratio (TBRmax), a measure of the point of most intense 18F-NaF uptake within the volume of interest, was calculated as the valvular maximum standardised uptake value (SUVmax) divided by the atrial blood pool mean standardised uptake value (SUVmean). TBRmean, a measure of the average ^18^F-NaF uptake in the volume of interest, was calculated as the valvular SUVmean divided by the blood pool SUVmean. Within the volume of interest, we measured the aortic valve microcalcification activity (AVMA), representing the overall disease activity in the aortic valve and based on both the volume and intensity of ^18^F-NaF PET activity within it (similar in principle to coronary microcalcification activity).^15^ AVMA was defined as the integrated activity in SUV units exceeding the corrected background blood pool mean SUV plus 2 SD (right atrium activity). CT aortic valve calcium (AVC) scoring according to Agatston methodology^16^ was performed using dedicated software on axial views (Syngo.via, Siemens, Medical Systems). All measurements were performed by experienced readers who were blinded to Lp(a) levels.

### Statistical analysis

Data are presented as mean±SD for normally distributed variables, median with IQR for non-normally distributed variables, and number (percentage) for categorical variables. Between group testing was performed using t-test for normally distributed data, Mann-Whitney U test for non-normally distributed data, and χ^2^ or Fisher’s exact test for categorical data. Based on previous PET/CT studies for the assessment of arterial wall inflammation,^17^ we conservatively estimated the expected mean difference in valvular ^18^F-NaF uptake between high and low Lp(a) groups to be 15%. To detect this difference with α=0.05% and 80% power, we aimed to enrol 25 patients per group. Multivariable models accounting for age, sex, AVC score and significantly different baseline clinical parameters (p<0.05) were used to assess differences in valvular TBR between high and low Lp(a) groups. Exploratory linear regression analyses were performed to assess whether increasing Lp(a) within the high Lp(a) group was associated with active calcification, and to evaluate if significantly different baseline characteristics (if any) were related to active calcification in the low Lp(a) group. AVC scores were log-transformed. A two-sided p value <0.05 was considered statistically significant. The statistical analyses were performed using RStudio software (V.4.0.3; R Foundation for Statistical Computing, Vienna, Austria).

### Patient and public involvement

Participants were not involved in determining the research question or outcome measures, nor were they involved in recruitment, design or implementation of the study. Participants were not asked for advice on the interpretation of results.

## Results

### Study population

In total, 58 patients were included between February 2018 and October 2020. One patient withdrew prior to PET/CT imaging, one was excluded from further analysis due to absence of aortic valve calcification, and one was excluded due to a history of radiation for breast cancer. In addition, we were unable to find an appropriate match for three participants. A total of 52 patients, consisting of 26 matched pairs, were included in the current analysis. There were no missing data. Baseline characteristics are listed in table 1. The mean age at the date of inclusion was 66.0±4.2 years. The majority of participants were men (86.7%). Five patients in each group had bicuspid valve morphology.

**Table 1:** Baseline characteristics

	High Lp(a) (n=26)	Low Lp(a) (n=26)	P value
Clinical characteristics			
Age, years	66.3±5.6	66.5±5.6	0.934*
Male gender	22 (84.6)	22 (84.6)	>0.999†
Body mass index, kg/m^2^	26.9±4.5	28.5±4.2	0.316*
Systolic blood pressure, mm Hg	128±12	141±12	<0.001*
Diastolic blood pressure, mm Hg	79±12	85±11	0.057*
Ischaemic heart disease	6 (23.1)	2 (7.7)	0.248‡
Hypertension	20 (76.9)	20 (76.9)	>0.999†
Diabetes mellitus	3 (11.5)	7 (26.9)	0.291‡
Smoking			
Never	8 (30.8)	9 (34.6)	0.872‡
Former	12 (46.2)	13 (50)	
Active	6 (23.1)	4 (15.4)	
Use of lipid-lowering therapy	19 (73.1)	16 (61.5)	0.554†
Use of antihypertensive(s)	18 (69.2)	19 (73.1)	>0.999†
Laboratory values			
C reactive protein, mg/dL	1.1 (0.5–4.8)	1.3 (0.9–2.3)	0.521*
Creatinine, mmol/L	87±20	87±18	0.903*
Urea, mmol/L	6.8±2.3	7.0±2.1	0.775*
Calcium, mmol/L	2.41±0.09	2.42±0.08	0.713*
Phosphate, mmol/L	0.94±0.20	0.94±0.16	0.933*
Total cholesterol, mmol/L	4.43±0.94	4.85±1.13	0.151*
HDL-cholesterol, mmol/L	1.54±0.34	1.62±0.72	0.606*
LDL-cholesterol, mmol/L	2.3±0.8	2.6±1.1	0.361*
Corrected LDL-cholesterol, mmol/L	1.9±0.8	2.5±1.3	0.035*
Triglycerides, mmol/L	1.09 (0.70–1.55)	1.29 (1.05–2.11)	0.090§
Lp(a), mg/dL	79 (64–112)	5 (3–10)	<0.001**§**
Echocardiographic parameters			
Bicuspid aortic valve	5 (19.2)	5 (19.2)	>0.999†
Peak aortic jet velocity, m/s	2.77±0.55	2.83±0.42	0.689*
Peak aortic valve gradient, mm Hg	31±13.55	30.85±10.68	0.964*
Mean aortic valve gradient, mm Hg	17.88±8.31	17.88±6.96	0.998*
Aortic valve area, cm^2^	1.77±0.64	1.62±0.42	0.345*

Data are presented as mean±SD, median (IQR) or number (percentage).

*Hypothesis tested by t-test.

†Hypothesis tested by χ^2^.

‡Hypothesis tested by Fisher’s exact test.

§Hypothesis tested by Mann-Whitney U test.

HDL, high-density-lipoprotein; LDL, low-density-lipoprotein; Lp(a), lipoprotein(a).

### Higher and lower Lp(a) groups

Higher (>50 mg/dL) and lower (<50 mg/dL) Lp(a) groups were well matched for age, sex, aortic valve morphology and peak aortic jet velocity (table 1). There was a more than tenfold difference in Lp(a) levels between the two groups. The median Lp(a) in the high group was 79 (IQR 64–117) mg/dL, compared with 7 (IQR 5–11) mg/dL in the lower Lp(a) group. Corrected LDL-C levels were significantly higher in the lower Lp(a) group (1.9±0.8 mmol/L in the high vs 2.5±1.1 mmol/L in the low Lp(a) group, p=0.035), as was systolic blood pressure (128±12 mm Hg in the high vs 141±12 mm Hg in the low Lp(a) group, p<0.001).

### Valvular imaging parameters

**Figure 1:**
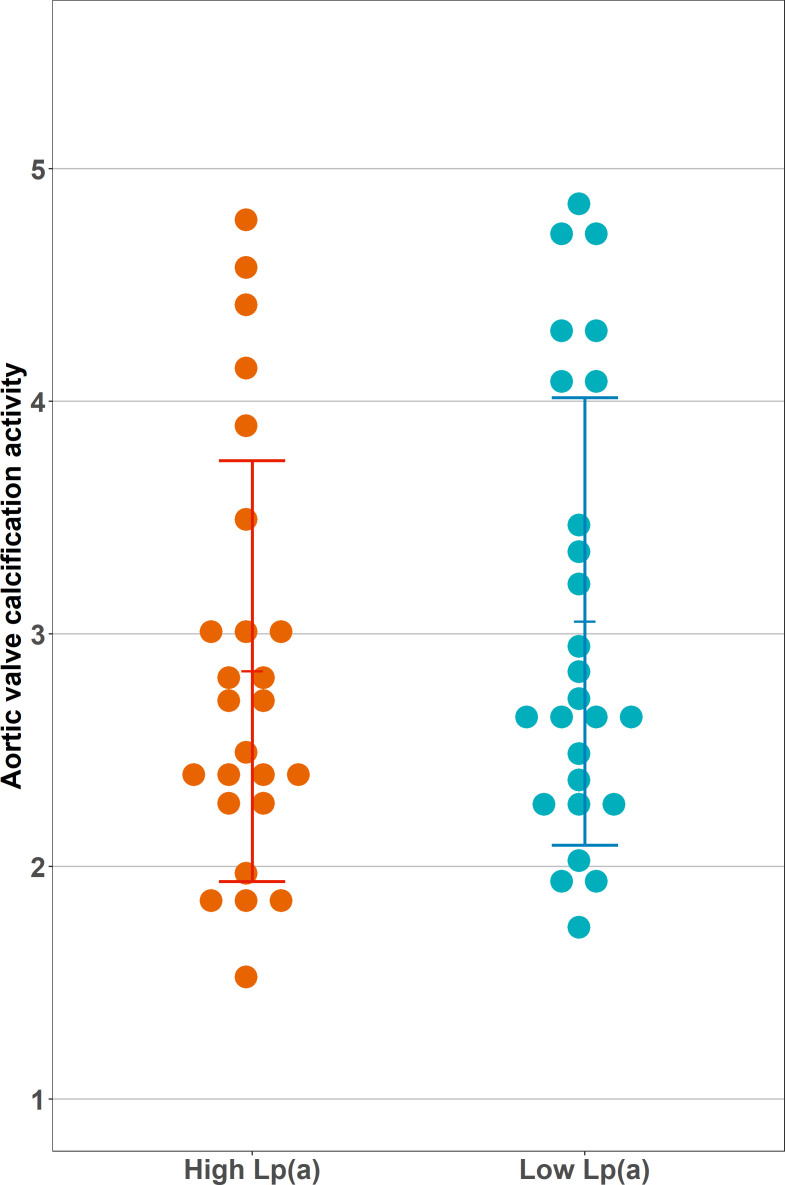
Aortic valve calcification activity stratified by lipoprotein(a) (Lp(a)) group. Depicted is the calcification activity of the aortic valve, measured as maximum target to background ratio. This was calculated by dividing the valvular maximum standardised uptake by the blood pool mean standardised uptake value. There was no significant difference in calcification activity between high and low Lp(a) groups (3.02±1.26 vs 3.05±0.96, p=0.902).

All valvular imaging parameters are listed in table 2. The CT AVC scores in the high and low Lp(a) groups were comparable between the groups (1388 Agatston units (AU) (450–2424) in the high vs 1173 (927–1628) AU in the low Lp(a) group, p=0.839). In contrast to our hypothesis, we did not observe a significant difference in calcification activity between the groups: the aortic valve TBRmax was 3.02±1.26 in the high Lp(a) group compared with 3.05±0.96 in the low Lp(a) group (p=0.902). The TBRmean showed comparable results, being 1.84±0.42 in the high Lp(a) group vs 1.86±0.49 in the low Lp(a) group (p=0.863). SUV values also did not differ significantly between the groups nor did AVMA. Figure 1 shows the distribution of valvular ^18^F-NaF uptake in both groups.

**Table 2:** Aortic valve imaging parameters

	High Lp(a) (n=26)	Low Lp(a) (n=26)	P value
Aortic valve calcium score (AU)	1388 (450–2424)	1173 (927–1628)	0.839*
Aortic valve TBRmax	3.02±1.26	3.05±0.96	0.902†
Aortic valve TBRmean	1.84±0.42	1.86±0.49	0.863†
Aortic valve SUVmax	3.29±1.38	3.10±0.79	0.553†
Aortic valve SUVmean	1.87±0.40	2.00±0.64	0.399†
Aortic valve microcalcification activity	8.30±3.58	6.96±2.68	0.144†
Right atrium SUVmean	1.08±0.32	1.04±0.24	0.640†

Data are presented as mean±SD or median (IQR).

*Hypothesis tested by Mann-Whitney U test.

†Hypothesis tested by t-test.

AU, Agatston units; Lp(a), lipoprotein(a); SUVmax, maximum standardised uptake value; SUVmean, mean standardised uptake value; TBRmax, maximum target to background ratio; TBRmean, mean target to background ratio.

### Determinants of valvular calcification activity

In univariable regression analysis, only AVC score and male sex were significantly associated with valvular ^18^F-NaF uptake, whereas age, Lp(a) group, LDL-C and systolic blood pressure were not. Univariable analysis with only CT AVC score as determinant of valvular ^18^F-NaF uptake showed a better fit than the multivariable model with age, sex, Lp(a) group, systolic blood pressure and corrected LDL-C (adjusted R^2^=0.40 for univariable and adjusted R^2^=0.35 for multivariable model). In multivariable linear regression analysis, the AVC score was the only determinant of valvular ^18^F-NaF uptake (β=0.63 per 1000 increase in AU, 95% CI 0.38 to 0.88, p<0.001). Lp(a) group was not significantly associated with valvular ^18^F-NaF uptake (β=0.17 for the high Lp(a) group, 95% CI −0.44 to 0.78, p=0.305), nor were age, sex, systolic blood pressure and corrected LDL-C (table 3). As an exploratory analysis, we assessed whether increasing Lp(a) was associated with more calcification activity in the high Lp(a) group only, and whether increasing blood pressure and LDL-C were drivers of calcification activity in the low Lp(a) group, but CT AVC score remained the only predictor (online supplemental table 1).

10.1136/heartjnl-2021-319804.supp1 Supplementary data

**Table 3:** Linear regression analysis for valvular ^18^F-NaF uptake

	Univariable analysis β (95% CI)	P value	Multivariable analysis β (95% CI)	P value
Intercept			2.65 (−2.65 to 6.23)	0.217
Aortic valve calcium score (per 1000 AU increase)	0.60 (0.39 to 0.81)	<0.001	0.63 (0.38 to 0.88)	<0.001
Age (per 10-year increase)	0.23 (−0.34 to 0.80)	0.416	−0.25 (−0.74 to 0.24)	0.305
Male sex	0.87 (0.04 to 1.70)	0.041	0.06 (−0.77 to 0.88)	0.891
High lipoprotein(a) group	0.04 (−0.59 to 0.66)	0.902	0.17 (−0.44 to 0.78)	0.573
Systolic blood pressure (per 10 mm Hg increase)	0.05 (−0.18 to 0.29)	0.639	0.10 (−0.12 to 0.32)	0.387
Corrected LDL-C (per mmol/L increase)	0.19 (−0.11 to 0.50)	0.216	−0.07 (−0.36 to 0.23)	0.648

Data are standardised coefficients (β) with 95% CI.

Adjusted R^2^=0.40 for the univariable analysis with aortic valve calcium score and adjusted R^2^=0.35 for the multivariable analysis.

AU, Agatston units; LDL-C, low-density-lipoprotein cholesterol; NaF, sodium fluoride.

## Discussion

**Figure 2:**
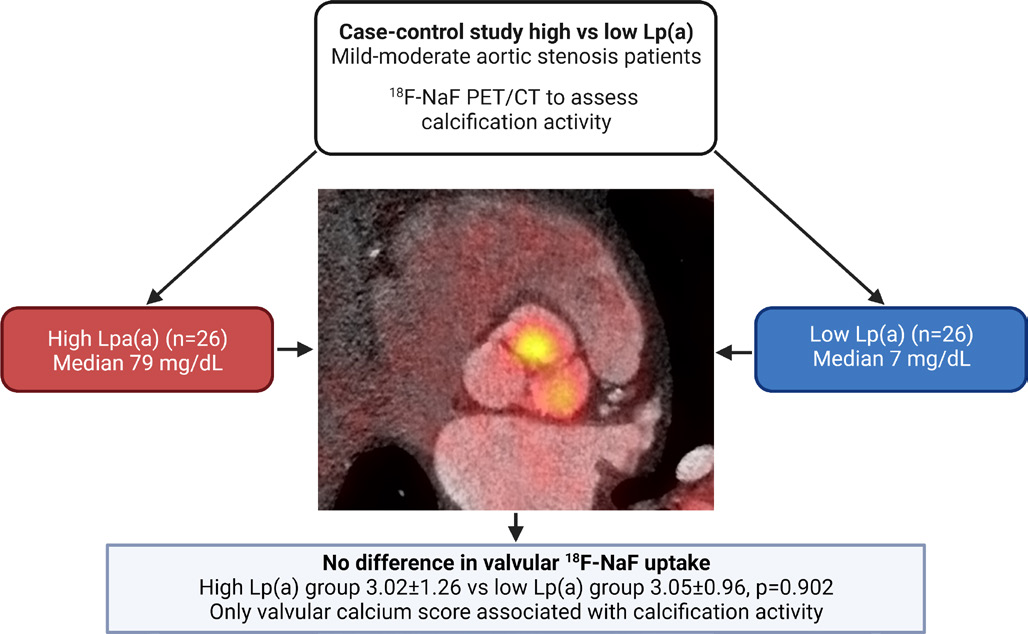
Lp(a) has no major impact on calcification activity in patients with mild to moderate aortic valve stenosis. In this case–control study consisting of matched patients with aortic stenosis with high versus low Lp(a), we observed comparable calcification activity in both groups. Aortic valve calcium score was the only variable associated with ^18^F-NaF uptake in linear regression analysis (β=0.60 per 1000 Agatston unit increase, 95% CI 0.39 to 0.81). Lp(a), lipoprotein(a); NaF PET/CT, sodium fluoride positron emission tomography/CT.

In our study, we did not find a significant difference in valvular calcification activity, assessed with ^18^F-NaF PET/CT, in patients with mild to moderate AS and higher levels of Lp(a), compared with patients with lower Lp(a), when matched for age, sex and AS severity (figure 2). Since patients with lower Lp(a) were characterised by a significant increase in systolic blood pressure and LDL-C, both known risk factors for AS, this may have obscured a potential effect of Lp(a). However, we observed the CT AVC score to be a highly significant predictor of valvular ^18^F-NaF uptake in patients with mild to moderate AS. These results suggest that initiating risk factors may lose their relative importance in more advanced stages of AS, which seems to be primarily driven by valvular calcific burden.

### Lp(a) and initiation of aortic valve disease

Lp(a) has been demonstrated to accumulate within the aortic valve leaflets of patients with AS, co-localising with calcified areas. Mechanistically, Lp(a) was shown to cause osteogenic differentiation in valvular interstitial cells via increased phosphorylation of several kinases such as p38 mitogen-activated protein kinase and glycogen synthase kinase 3 beta.^18^ In support of a potent calcification response of Lp(a), Després and colleagues^19^ observed higher valvular ^18^F-NaF uptake in healthy individuals with high Lp(a) levels of whom the majority had no observable calcifications on CT. In our study, valvular ^18^F-NaF uptake was approximately twofold higher than in the healthy volunteers in the study by Després and colleagues,^19^ but we did not observe a difference in ^18^F-NaF uptake between patients with markedly elevated versus low Lp(a) levels. The discrepancy between these studies likely relates to the presence of established calcifications in the aortic valves of all our patients as opposed to none to negligible calcifications in Després and colleagues’^19^ study. In support, CT AVC score was highly predictive of ^18^F-NaF uptake in patients with AS, implying that the contribution of Lp(a) is lower in more advanced disease stages of AS.^20^

### Lp(a) and progression of established AS

In previous studies, Lp(a) has also been associated with faster AS progression. This was first demonstrated in a post-hoc analysis of the Aortic Stenosis Progression Observation: Measuring Effects of Rosuvastatin (ASTRONOMER) trial, which evaluated the effect of statin treatment in relatively young patients (mean age 58 years) with mild to moderate AS.^21^ Elevated Lp(a) levels were associated with more rapid haemodynamic AS progression on echocardiography over a median follow-up of 3.5 years, especially in younger patients (<57 years, median). These data suggested that as patients get older, other risk factors or disease mechanisms may come into play as drivers of disease progression. A subsequent post-hoc analysis of two longitudinal imaging studies (Scottish Aortic Stenosis and Lipid Lowering Trial, Impact on Regression (SALTIRE)/Ring of Fire) also supported a role of Lp(a) in older patients (mean age 70 years) with more advanced AS.^9^ Patients in the top Lp(a) tertile (>35 mg/dL) showed higher valvular ^18^F-NaF uptake at baseline, which associated with faster CT AVC score progression and a higher HR for valve replacement and cardiovascular disease during follow-up, as compared with patients in the lower tertiles of Lp(a). Although we selected patients with mild to moderate AS and much higher Lp(a) levels in this study, we could not establish a difference in ^18^F-NaF uptake between high and low Lp(a) patients.

### What drives progression of established aortic valve disease?

After strict matching for AS severity, age and sex, we did observe significantly higher blood pressure and LDL-C in patients with AS with low Lp(a). It is possible that the significantly higher blood pressure and LDL-C in the low Lp(a) group obscured the effect of Lp(a) on valvular disease activity. Nevertheless, we did not find associations between blood pressure, LDL-C and valvular ^18^NaF uptake. Additionally, we did not find increasing Lp(a) levels within the high Lp(a) group to be associated with increased calcification activity, nor were systolic blood pressure or LDL-C associated with calcification activity in the low Lp(a) group. The CT AVC score was the only determinant of valvular calcification activity, even though we included patients with milder disease (peak aortic jet velocity 2.8 m/s) as compared with the previous imaging studies. While at odds with the Lp(a) literature to date, this observation is consistent with other data demonstrating that while traditional cardiovascular risk factors (eg, LDL-cholesterol) are associated with new-onset incident AVC, they show no relation with disease progression after correction for baseline AVC score.^22 23^ Further studies are now required to resolve this apparent discrepancy and also whether the relationship between Lp(a) and disease progression depends on baseline AS severity.

### Study limitations

This study has several limitations that deserve consideration. First, our sample size was small and only able to detect a 15% or larger difference in valvular ^18^F-NaF uptake between groups. This modest sample size also led to the linear regression analyses having more predictors than would be appropriate for the number of observations. However, as the beta for the high Lp(a) group in our linear regression analysis was only 0.17, which reflects 5.6% of total valvular ^18^F-NaF uptake, it is unlikely that a larger sample size would have been able to show a significant relationship between Lp(a) and calcification activity. Our study does not refute that Lp(a) causes calcification, which has been clearly established, but its effect in mild to moderate AS may be less pronounced than initially expected. Another important limitation was the higher burden of competing risk factors (systolic blood pressure and LDL-C) in the low Lp(a) group. Our study was designed to study the intrinsic impact of Lp(a) on ^18^F-NaF uptake, and therefore we applied very strict matching on age, sex and AS severity. Apparently, patients with low Lp(a) required a higher burden of alternative risk factors in order to achieve similar AS severity, which may have masked an association between Lp(a) and ^18^F-NaF uptake. However, the robust relationship between CT AVC score and valvular ^18^F-NaF uptake, combined with the absence of an association between Lp(a), blood pressure, LDL-C and calcification activity, makes this less likely. Nevertheless, we recognise that our study did not have sufficient power to adequately investigate the effects of these competing risk factors on calcification activity. Finally, we do not have data on the progression of valve calcification in the current study, which is currently a better validated endpoint than ^18^F-NaF PET.

## Conclusion

Among patients with mild to moderate AS, we observed that valvular ^18^F-NaF uptake, a marker of calcification activity, is predominantly determined by established calcium burden. The results do not support our hypothesis that Lp(a) is associated with valvular ^18^F-NaF uptake. Nevertheless, due to the unexpected imbalance of competing risk factors in our study population, we cannot rule out that Lp(a) propagates calcification activity in patients with AS with high Lp(a) levels. Our results imply that the established valvular calcific burden is the most important disease driver of AS. Further research evaluating the contribution of Lp(a) at different stages of AS severity is warranted.

**Key messages**

**What is already known on this subject?**

- There is substantial evidence that lipoprotein(a) (Lp(a)) is a causal risk factor for incident aortic valve stenosis (AS).
- Recent post-hoc analyses suggest that Lp(a) also accelerates AS progression.

**What might this study add?**

- Contrary to our hypothesis, elevated Lp(a) was not associated with a higher degree of calcification activity measured with ^18^F-sodium fluoride positron emission tomography/CT.
- The only driver of calcification activity was valvular Agatston score.
- These data suggest that the association between Lp(a) and disease progression may depend on the severity of aortic stenosis as well as other competing risk factors.

**How might this impact on clinical practice?**

- The absence of an effect of Lp(a) on calcification activity in mild to moderate AS may require a shift in focus towards aggressive Lp(a)-lowering in patients with profound Lp(a) elevation prior to the onset of AS.

## Data Availability

No data are available.
