# Supplementary material for: Lipoprotein(a) has no major impact on calcification activity in patients with mild to moderate aortic valve stenosis
Source: Heart. 2021 Sep 30;108(1):61–6. doi: 10.1136/heartjnl-2021-319804 (PMC8666821; doi:10.1136/heartjnl-2021-319804)
Supplement: Supplementary data [file heartjnl-2021-319804supp001.pdf]

Supplementary Table 1. Relationship between Lp(a), systolic blood pressure, LDL-C and valvular ^{18}F-NaF uptake

**High Lp(a) group (n=26)**				
	Univariate analysis		Multivariable analysis	
	β (95%CI)	p value	β (95%CI)	p value
Intercept			2.09 (1.14, 3.04)	< 0.001
Aortic valve calcium score (per 1,000 AU increase)	**0.76 (0.48, 1.04)**	**< 0.001**	**0.75 (0.47, 1.04)**	**< 0.001**
Lp(a) (per 50 mg/dL increase)	-0.20 (-0.76, 0.36)	0.458	-0.11 (-0.50, 0.27)	0.540
**Low Lp(a) group (n=26)**				
	Univariate analysis		Multivariable analysis	
	β (95%CI)	p value	β (95%CI)	p value
Intercept			0.45 (-4.09, 4.99)	0.839
Aortic valve calcium score (per 1,000 AU increase)	**0.39 (0.07, 0.71)**	**0.020**	**0.39 (0.03, 0.75)**	**0.034**
Systolic blood pressure (per 10 mmHg increase)	-0.03 (-0.36, 0.31)	0.859	0.12 (-0.20, 0.43)	0.442
Corrected LDL-C (per mmol/L increase)	0.33 (-0.01, 0.66)	0.054	0.11 (-0.25, 0.47)	0.534

Data are standardized coefficients (β) with 95% confidence intervals (CI). Lp(a) = lipoprotein(a), NaF = sodium-fluoride, AU = Agatston units, LDL-C = low-density-lipoprotein cholesterol.
